# Hepatoprotection by Methylene Blue Against Doxorubicin Toxicity Through Coordinated Modulation of Oxidative Stress, ER Stress, and Apoptotic Pathways

**DOI:** 10.3390/ph18111625

**Published:** 2025-10-28

**Authors:** Enas S. Gad, Ahmed M. Ashour, Amany M. Gad, Ali Khames, Shaimaa G. Ibrahim, Mohamed H. A. Gadelmawla, Mona Mansour

**Affiliations:** 1 Department of Pharmacology and Toxicology, Faculty of Pharmacy, Sinai University, Kantara Branch, Ismailia 41636, Egypt; enasgad1988@gmail.com (E.S.G.); amany.gad@su.edu.eg (A.M.G.); 2 Department of Pharmaceutical Sciences, College of Clinical Pharmacy, King Faisal University, Al-Ahsa 13889, Saudi Arabia; 3 Department of Pharmacology and Toxicology, College of Pharmacy, Umm Al-Qura University, P.O. Box 13578, Makkah 21955, Saudi Arabia; 4 Department of Pharmacology, Egyptian Drug Authority (EDA)-Formerly NODCAR, Giza 12654, Egypt; 5 Department of Pharmacology and Toxicology, Faculty of Pharmacy, Sohag University, Sohag 82511, Egypt; ali.khames@pharm.sohag.edu.eg; 6 Department of Pharmacology and Toxicology, Faculty of Pharmacy, October 6 University, Giza 12585, Egypt; drshaimaa.gomaa@yahoo.com; 7 Department of Life Sciences, Faculty of Biotechnology, Sinai University, Kantara Branch, Ismailia 41636, Egypt; 8 Department of Pharmacology and Toxicology, Faculty of Pharmacy (Girls), Al-Azhar University, Cairo 11884, Egypt; mona.mansour@azhar.edu.eg

**Keywords:** methylene blue, hepatotoxicity, doxorubicin, inflammation, endoplasmic reticulum stress, apoptosis

## Abstract

**Background and Objectives:** Doxorubicin (DOX) is a potential chemotherapeutic whose clinical application is limited by hepatotoxicity mediated through apoptosis, endoplasmic reticulum (ER) stress, and oxidative stress (OS). This study aimed to assess the hepatoprotective impact of methylene blue (MB) against DOX-induced liver injury. **Methods:** Forty rats were arbitrarily divided equally into four groups: control, DOX (15 mg/kg, i.p., single dose), MB (4 mg/kg, i.p., daily for 7 days), and DOX + MB (same regimen, MB initiated 1 h post DOX). Serum ALT, AST, and γ-GT were measured, along with hepatic TAC and HO-1. ELISA quantified PERK, GRP78, and CHOP. Immunohistochemistry assessed Caspase-3, p53, NF-κB, and Nrf2. Histopathological evaluation was performed using H&E staining. **Results:** DOX administration significantly elevated ALT, AST, γ-GT, HO-1, PERK, GRP78, and CHOP while reducing TAC and Nrf2 expression. Strong Caspase-3, p53, and NF-κB immunoreactivity and severe histopathological damage were observed. MB treatment markedly reversed these changes, restoring antioxidant status, downregulating ER stress markers, preserving Nrf2 expression, and improving hepatic architecture. **Conclusions:** MB exerts significant hepatoprotection against DOX-induced injury, likely via attenuation of OS, ER stress, apoptosis, and inflammation.

## 1. Introduction

Doxorubicin (DOX) is a key chemotherapeutic agent with strong effectiveness towards hematological and various solid tumors [1]. Nevertheless, DOX utilization clinically is limited due to adverse impacts on multiple organs, including the bone marrow, intestinal epithelium, heart, liver, and kidneys, as well as the potential for inducing cancer cell resistance [2]. Both powdered and liquid versions of DOX are accessible, and it is usually given intravenously [3]. The administration routes of both doxorubicin (intraperitoneal) and methylene blue (intraperitoneal) were selected based on their established efficacy in preclinical rodent models. While intravenous administration is the standard in human clinical practice, the intraperitoneal route provides a reliable and practical alternative for achieving systemic exposure in rats, ensuring translational relevance for early-stage mechanistic studies. Despite its potent chemotherapeutic activity, doxorubicin’s conventional application has been reduced due to serious adverse impacts on different organs, leading to fertility issues, nephro-, cardio-, and hepatotoxicity, and the initiation of diabetic cardiac injury [4,5,6]. Its biphasic toxicity results in both acute and sub-acute hepatic damage [7]. The generation of reactive oxygen species (ROS) during DOX metabolism in the liver, causing an imbalance in redox potential, is the main molecular mechanism behind DOX-induced hepatotoxicity (DIHT). Oxidative stress (OS), reduced antioxidant enzyme values, apoptosis, inflammation, and mitochondrial malfunction are caused by this imbalance. Metabolism of doxorubicin in the liver generates superoxide radicals and peroxynitrite radicals, initiating lipid peroxidation and causing hepatic damage, evidenced by the production serum AST and ALT (hepatic enzymes). These enzyme levels serve as biomarkers for hepatotoxicity [8,9]. The drug also depletes antioxidant molecules like glutathione peroxidase (GPx), catalase (CAT), superoxide dismutase (SOD), and glutathione (GSH), thereby halting the activity of defensive mechanism [10,11]. Genes that produce apoptotic enzymes like Caspase-3 and antioxidant enzymes like Nrf2 and HO-1 are elevated within DOX therapy. ROS are produced when malondialdehyde values are elevated, leading to a cascade of apoptotic events [8,12]. The lipophilic nature of DOX and its DNA-binding capacity contribute to its capacity to accumulate in hepatocytic nuclei, leading to DNA damage [13]. The hepatotoxicity caused by DOX has been lessened by several drugs, according to a number of studies. It has been demonstrated that a number of natural products and medications have hepatoprotective properties that lessen the negative effects of DOX.

Methylene blue (MB), a thiazine dye, is utilized in tissue staining, histological studies, and medical interventions [14]. It is effective in treating conditions such as encephalopathy [15], methemoglobinemia, and poisonings caused by carbon monoxide, cyanide, and nitrates [16]. Additionally, MB helps prevent septic shock hypotension [17], and protects against renal and hepatic injury [18]. It also acts as a bacteriostatic disinfectant for the genitourinary tract [19]. Among its notable therapeutic properties is its impact on central nervous system (CNS) conditions. Furthermore, its effects on liver diseases, kidney disorders, lung damage, and cardiovascular conditions have been explored [20]. MB is FDA-approved for treating methemoglobinemia of various origins [21]. One of its most intriguing characteristics is its influence on mitochondrial function and regulation and ROS generation [15]. This study aims to assess the mechanisms by which methylene blue can mitigate liver damage induced by DOX, with a focus on the roles of OS and apoptosis.

## 2. Results

### 2.1. Effect of MB on DOX-Induced Hepatic Dysfunction

**Figure 1:**
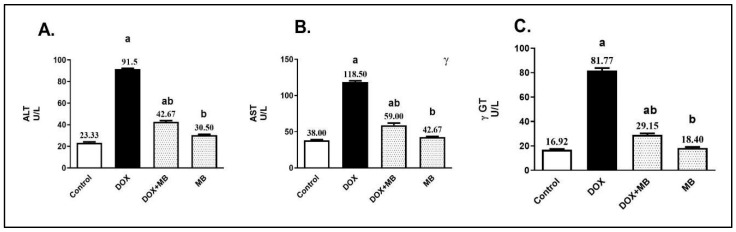
MB effects on serum ALT (**A**), AST (**B**), and γ-GT (**C**) levels. Data are expressed as mean ± S.E.M (n = 10). a: significance towards control, b: significance towards DOX group at *p* < 0.05. Abbreviations: ALT, alanine aminotransferase; AST, aspartate aminotransferase; γ-GT, gamma-glutamyl transferase; DOX, doxorubicin; S.E.M., standard error of the mean.

The serum levels of alanine aminotransferase (ALT), aspartate aminotransferase (AST), and gamma-glutamyl transferase (γ-GT) were ultimately upregulated in the DOX group (15 mg/kg, IP, once) by 3.9-, 3.1-, and 4.8-fold, respectively, compared to the control group (Figure 1). MB-treated rats (4 mg/kg/IP/daily for 7 days) exhibited a remarkable decline in liver enzyme values by 53.4%, 50.2%, and 64.4%, respectively, in comparison to the DOX group (*p* < 0.05).

### 2.2. MB Effects on Hepatic OS Markers Against DIHT

**Figure 2:**
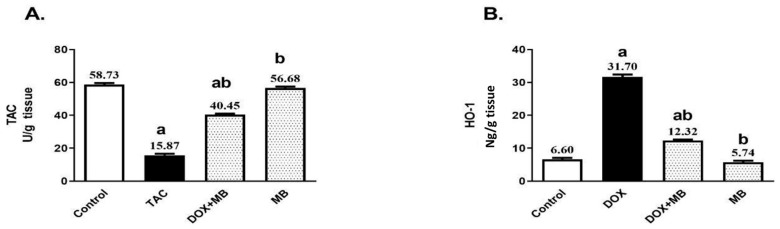
Effects of MB on total antioxidant capacity (TAC) (**A**) and HO-1 (**B**) contents. Data are expressed as mean ± S.E.M (n = 10). a: significance towards control, b: significance towards DOX group at *p* < 0.05. Abbreviations: TAC, total antioxidant capacity; HO-1, heme oxygenase-1; DOX, doxorubicin; S.E.M., standard error of the mean.

The oxidative stress (OS) impact of MB against DOX-induced ROS production was evaluated by measuring the contents of total antioxidant capacity (TAC) and heme oxygenase-1 (HO-1). Induction of DOX hepatotoxicity provided a significant decrease in hepatic TAC by about 73%, as well as a significant increase in hepatic HO-1 by 4.8-fold compared to the control group (Figure 2). MB-treated rats revealed a remarkable elevation in the hepatic content of TAC by 154.9% and prevented the elevation in HO-1 content by about 61.1% compared to the DOX group (*p* < 0.05).

### 2.3. Effects of MB on PERK/GRP78/CHOP Pathway Markers

**Figure 3:**
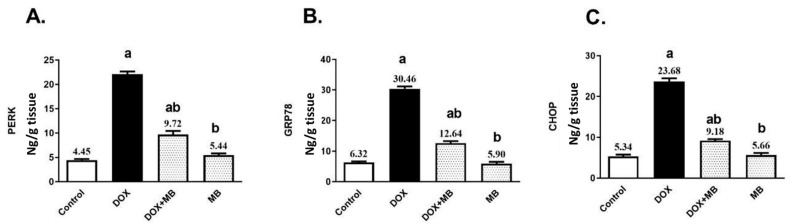
MB effects on PERK (**A**), GRP78 (**B**), and CHOP (**C**) contents. Data are expressed as mean ± S.E.M (n = 10). a: significance towards control, b: significance towards DOX group at *p* < 0.05. Abbreviations: DOX, doxorubicin; PERK, protein kinase RNA-like endoplasmic reticulum kinase; GRP78, glucose-regulated protein 78; CHOP, C/EBP homologous protein; S.E.M., standard error of the mean.

Doxorubicin (DOX) substantially elevated the hepatic contents of PERK, GRP78, and CHOP by about 5-, 4.8- and 4.4-fold, respectively, compared to the control group, as presented in Figure 3. Treatment with MB revealed a remarkable reduction in the hepatic contents of PERK, GRP78, and CHOP by 56.06%, 58.5%, and 61.23%, respectively, in comparison to the DOX group (*p* < 0.05).

### 2.4. Evaluation of Histological Changes

**Figure 4:**
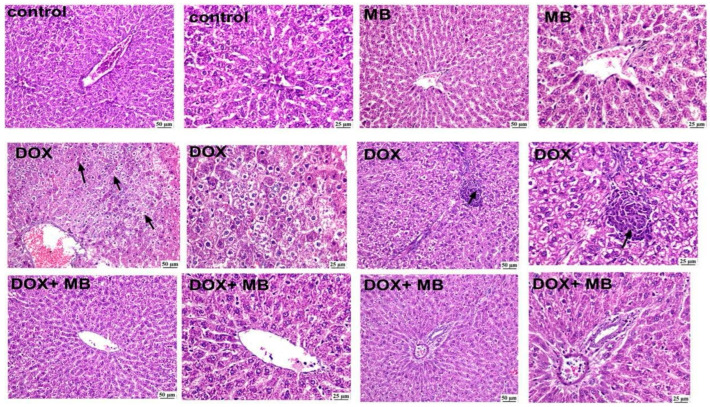
The photomicrographs show the liver tissue of all groups. The control group displayed normal hepatic architecture with well-arranged hepatocyte cords radiating from the central vein, intact cytoplasm, and clearly defined nuclei. The MB group revealed apparently normal hepatocytes in the centrilobular and periportal areas, indicating no morphological alterations. In contrast, the DOX group exhibited marked hepatic injury characterized by diffuse hepatocellular vacuolation, cytoplasmic degeneration, and focal necrosis accompanied by mononuclear inflammatory cell infiltration (arrows). The DOX + MB group demonstrated a remarkable improvement in hepatic architecture, showing nearly normal hepatocytes around the central and portal areas, with restoration of cellular integrity and minimal vacuolar degeneration (100 and 400×, H&E). Abbreviations: DOX, doxorubicin; H&E, hematoxylin and eosin.

Histopathological examinations from the normal control group (Figure 4) revealed normal histologic structure of hepatic parenchyma in both centrilobular and portal areas. Likewise, apparently normal liver sections were detected in the MB-alone group. The DOX group exhibited marked histopathological alterations, and excessive hepatocellular vacuolation was noticed. Focal areas of necrosis with inflammatory cell infiltration were detected. The portal areas were heavily infiltrated with mononuclear inflammatory cells. Concerning the DOX + MB group marked improvement was detected, as hepatocytes in the centrilobular areas were apparently normal with mild mononuclear inflammatory cell infiltration in the portal areas.

### 2.5. Evaluation of Immunohistochemistry Changes

**Figure 5:**
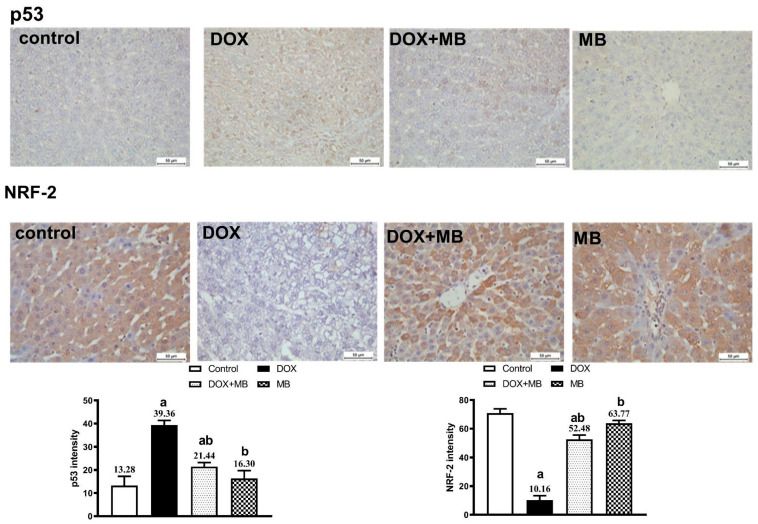
Protective effect of MB on hepatic immunoreactivity of p53 and NRF-2 in DOX-evoked hepatic damage in rats. Photomicrographs of liver tissues from all groups. The immunoreactivity of P53 and NRF-2 were visible in the tissues as a brown color generated by DAB chromogen (DAB, ×400). The control group exhibited normal hepatic histoarchitecture with faint p53 and moderate NRF-2 staining. The MB-alone group showed mild NRF-2 nuclear localization and negligible p53 expression. The DOX-treated group revealed intense p53 immunostaining in hepatocyte nuclei and cytoplasm, accompanied by a marked reduction in NRF-2 expression. In contrast, the DOX + MB group displayed attenuated p53 staining and enhanced NRF-2 immunoreactivity. Quantitative analysis of immunostaining intensity for p53 and NRF-2 is expressed as mean ± S.E.M. (n = 10). a: significant difference compared with the control group; b: significant difference compared with the DOX group at *p* < 0.05. Abbreviations: NRF-2, nuclear factor erythroid 2-related factor 2; DAB, 3,3′-diaminobenzidine; S.E.M., standard error of the mean.

**Figure 6:**
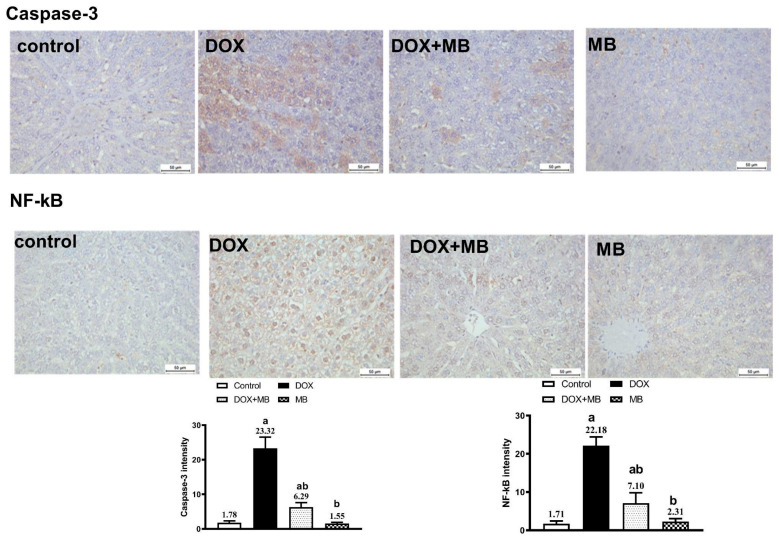
Protective effect of MB on hepatic immunoreactivity of Caspase-3 and NF-κB in DOX-treated rats evoked hepatic damage in rats. Photomicrographs of hepatic tissues of all groups. Caspase-3 and NF-κB reactivity were detected in tissues using DAB chromogen, resulting in a brown color (DAB, ×400). The control group exhibited weak cytoplasmic Caspase-3 and NF-κB staining. The MB-alone group displayed minimal immunoreactivity similar to the control. In contrast, the DOX group showed strong Caspase-3 and NF-κB staining. The DOX+MB group demonstrated markedly reduced immunostaining intensity for both markers. Quantitative analysis of Caspase-3 and NF-κB immunoreactivity is expressed as mean ± S.E.M. (n = 10). a: significant difference compared with the control group; b: significant difference compared with the DOX-treated group at *p* < 0.05. Abbreviations: NF-κB, nuclear factor kappa B; DAB, 3,3′-diaminobenzidine; S.E.M., standard error of the mean.

The IHC analysis demonstrated weak expression of Caspase-3, P53, and NF-κB in hepatic tissues of the control and MB groups. The DOX group showed severe expressions of Caspase-3, P53, and NF-κB. The DOX + MB group revealed a weak Caspase-3, P53, and NF-κB immunoreactivity approximately like the control tissues. However, for NRF-2, severe immunoreactivity was shown in the control and MB groups, moderate expression in the DOX + MB group, and weak expression in the DOX group (Figure 5 and Figure 6).

## 3. Discussion

This study has certain limitations. First, the use of the intraperitoneal route for drug administration may limit the translational relevance of the findings, and future studies employing intravenous delivery are recommended. Second, the analysis was restricted to a specific set of biomarkers, while inclusion of additional antioxidant markers such as SOD and catalase could provide a more comprehensive understanding. Finally, reliance on ELISA and immunohistochemistry without complementary molecular techniques, such as Western blotting or qPCR, limits mechanistic validation; thus, future work will aim to incorporate these methods to strengthen the molecular evidence.

Doxorubicin (DOX) is a widely prescribed anticancer agent with proven efficacy against numerous solid as well as hematological malignancies [22]. Its therapeutic usage is markedly limited by its dose-dependent and cumulative hepatotoxicity [23]. In certain cases, the hepatic damage provoked by DOX can become a serious clinical concern, potentially compromising the patient’s tolerance to further chemotherapy. DIHT is well documented in preclinical models. Among the principal mechanisms implicated, ER stress has a pivotal role. The substantial amount of unfolded/misfolded proteins inside the ER lumen during DOX exposure disrupts cellular homeostasis, initiating a cascade of signaling events that culminate in hepatocyte dysfunction and cell death [24,25].

The timing of methylene blue (MB) administration is crucial for optimizing its cardioprotective effects. In this study, MB was co-administered with doxorubicin (DOX), but whether pre- or post-treatment would yield similar benefits remains to be explored. Pre-treatment may enhance cellular defenses before DOX injury, while post-treatment better simulates clinical scenarios. Further studies comparing these strategies could improve the translational relevance of MB.

In the present study, DOX administration produced a clear biochemical signature of hepatocellular damage, elucidated by marked elevations in serum ALT, GGT, and AST, and this agreed with Gotama et al. [26] and Wu et al. [27]. These changes indicate loss of hepatocyte membrane integrity, mitochondrial perturbation, and oxidative stress-related impairment of hepatic function. Mechanistically, such elevations are consistent with DOX-induced ROS generation via redox cycling, which triggers lipid peroxidation and disrupts phospholipid bilayers. In addition, ER stress and disturbances in protein folding and calcium homeostasis further compromise cellular stability, facilitating enzyme leakage into the circulation. The observed pattern of increased ALT, AST, and GGT therefore reflects the combined effects of oxidative damage, ER and mitochondrial dysfunction, and subsequent hepatocyte necrosis/apoptosis, findings supported by the histopathological alterations detected in the DOX group [28].

In this study, DOX administration induced a marked oxidative imbalance, as evidenced by a pronounced reduction in hepatic TAC together with a substantial increase in HO-1 content, and this aligned with Barakat et al. [8] and Saleh et al. [29,30]. The decline in TAC reflects depletion of the endogenous antioxidant reserve, indicating that the hepatic defense system was overwhelmed by excessive ROS production. The elevation in HO-1 represents an adaptive cellular stress response, as this inducible enzyme is upregulated under oxidative challenge to degrade pro-oxidant heme and generate cytoprotective molecules. However, persistent or exaggerated HO-1 induction may also signify ongoing oxidative injury rather than successful adaptation. Together, the decrease in TAC and the rise in HO-1 demonstrate that DOX triggers severe oxidative stress in hepatic tissue, disrupting redox [30,31].

In the present study, HO-1 was selected as a representative antioxidant marker due to its dual role as both an inducible antioxidant enzyme and a cytoprotective mediator under oxidative stress conditions. HO-1 is particularly responsive to redox imbalance and has been shown to be upregulated in cardiac tissue following doxorubicin-induced injury, making it a sensitive indicator of oxidative stress modulation. While we acknowledge the established roles of other classical antioxidant enzymes such as superoxide dismutase (SOD), catalase (CAT), glutathione peroxidase (GPx), and peroxiredoxins (Prx), the scope of the current study was limited to evaluating early signaling responses, with HO-1 serving as a key target. Future studies are warranted to provide a more comprehensive profile of the antioxidant defense system by including a panel of enzymatic markers to fully delineate the redox-modulating effects of methylene blue.

**Figure 7:**
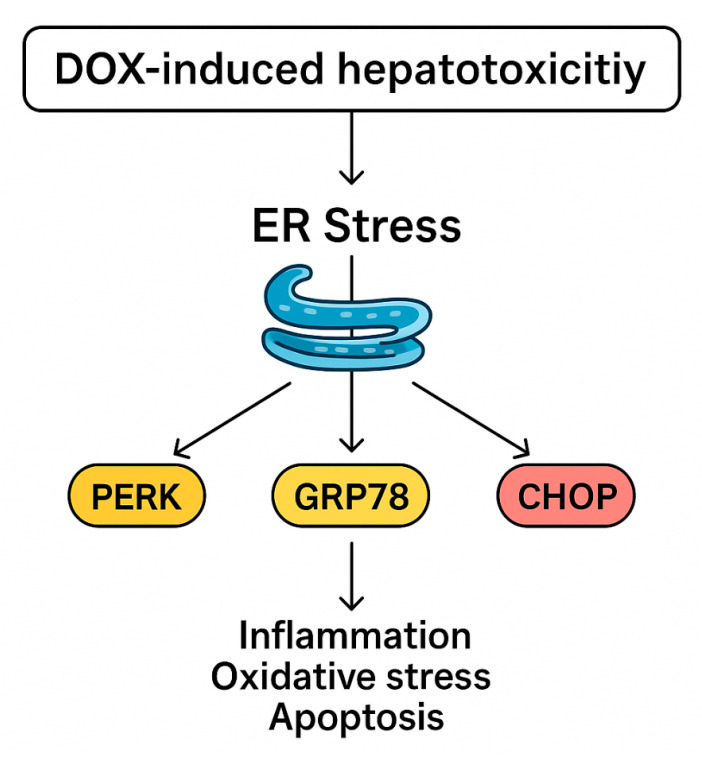
Proposed mechanism of DIHT.

In the present work, DOX administration markedly activated ER stress signaling, as demonstrated by significant increases in hepatic PERK, GRP78, and CHOP, and this is in accord with Yarmohammadi et al. [32] and Kopsida et al. [33]. PERK is a key transducer of the unfolded protein response, and its activation reflects ER matrix accumulated misfolded proteins. GRP78, an ER chaperone, is upregulated to stabilize protein folding and mitigate stress, while CHOP is a downstream transcription factor that shifts the ER stress response towards apoptosis when stress is severe or prolonged [34,35]. The concurrent elevation of these markers in DOX-treated animals indicates sustained ER stress that has transitioned from adaptive to pro-apoptotic signaling. Such activation is known to exacerbate hepatocellular injury by promoting inflammatory responses, amplifying oxidative stress, and triggering apoptotic pathways, thereby contributing to the overall hepatotoxic profile of DOX, as shown in Figure 7.

In the present study, immunohistochemical assessment provided further insight into the mechanistic pathway underlying DIHT. Hepatic tissues from DOX-intoxicated animals exhibited strong immunoreactivity for Caspase-3 and p53, indicating activation of the apoptotic pathway, and this is in agreement with Lin et al. [36] and Khafaga and El-Sayed [37]. p53 acts as a central tumor suppressor protein that responds to DNA damage by initiating pro-apoptotic signaling, while Caspase-3 serves as a key executioner enzyme that orchestrates the final stages of programmed cell death [38,39,40]. The concurrent overexpression of NF-κB in the DOX group reflects an associated inflammatory response, as it regulates the generation of numerous pro-inflammatory mediators and can further amplify tissue injury. In contrast, Nrf2 immunoreactivity was markedly reduced in the DOX group, consistent with impaired stimulation of the intracellular antioxidant defense [41]. The suppression of Nrf2 compromises transcriptional induction of cytoprotective genes, thereby exacerbating oxidative and inflammatory damage. Together, these findings confirm that DOX-induced hepatic injury is mediated by a combination of apoptosis, inflammation, and diminished antioxidant defense capacity [42,43].

The observed pattern of Nrf2 expression—high in the control and MB-treated groups, suppressed in the DOX group, and partially restored with DOX + MB co-treatment—is consistent with the redox-sensitive regulation of this transcription factor. Under basal conditions, Nrf2 contributes to cellular homeostasis by regulating antioxidant gene expression. DOX-induced oxidative stress is known to impair the Nrf2 pathway, leading to reduced nuclear translocation and degradation of the protein. Co-administration of MB likely alleviates this oxidative burden, stabilizing Nrf2 and enabling its partial activation, which contributes to the restoration of downstream antioxidant responses, including TAC. This biphasic response of Nrf2 under oxidative stress is well documented.

Histopathological analysis revealed normal hepatic architecture in both the control and MB groups. DOX-treated livers showed diffuse hepatocellular vacuolation, focal necrosis, and dense mononuclear inflammatory infiltration in portal areas, consistent with severe oxidative and ER stress-mediated injury. These changes reflect combined cytotoxic, metabolic, and inflammatory mechanisms underlying DIHT.

In the present study, MB co-administration markedly reduced the DOX-induced elevations in ALT, AST, and GGT, indicating effective preservation of hepatocyte integrity and function. This improvement suggests that MB was able to limit the extent of cellular membrane disruption and enzyme leakage into the circulation. The normalization of these hepatic enzyme levels reflects attenuation of DOX-mediated hepatocellular damage and supports the hepatoprotective role of MB in maintaining biochemical markers of liver function within near-normal ranges.

MB counteracts DOX-induced hepatic injury by restoring the antioxidant defense system, replenishing TAC, and reducing the oxidative burden signaled by HO-1 overexpression. With a strengthened redox balance, the excessive ROS generation that fuels cellular damage is curtailed, preserving intracellular homeostasis, and this is endorsed by Poteet et al. [44]. This reduction in oxidative stress dampens ER stress signaling, leading to lower activation of PERK, reduced expression of the chaperone GRP78, and suppression of the pro-apoptotic factor CHOP [45].

The absence of a significant antioxidant response following MB treatment alone suggests that its redox-modulating effects are conditionally activated under oxidative stress states. MB likely functions as a redox sensor or stress-responsive modulator, exerting its antioxidant potential primarily in environments of elevated ROS, such as those induced by DOX. In the absence of oxidative insult, endogenous antioxidant systems remain balanced, and MB does not significantly alter basal redox homeostasis. This context-dependent activity highlights MB’s potential selectivity in targeting pathological oxidative stress without disrupting normal cellular function, which is a favorable characteristic for therapeutic use.

**Figure 8:**
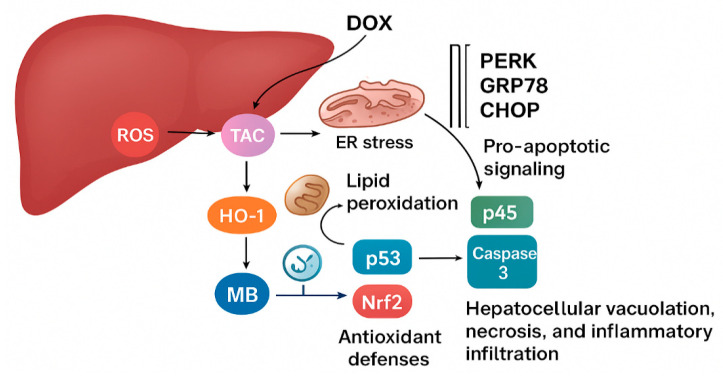
The proposed mechanism of MB’s hepatoprotective effect.

As the ER stress cascade is restrained, the downstream triggers of hepatocellular apoptosis and inflammation are weakened. The apoptotic signals driven by p53 and Caspase-3 diminish, while inflammatory amplification via NF-κB is blunted. At the same time, Nrf2 activity is partially restored, allowing sustained transcription of antioxidant and cytoprotective genes. Through this sequence of interlinked events beginning with oxidative stress suppression, progressing through ER stress modulation, and culminating in reduced apoptosis and inflammation [46], MB preserves hepatocyte structure and function, protecting the liver from progressive injury, as shown in Figure 8.

## 4. Materials and Methods

### 4.1. Experimental Animals and Design

Forty adult male albino rats (160–180 g) were purchased from the laboratory animal unit (NODCAR, Egypt). The rats received a standard control water and diet ad libitum for 7 days to become used to their new environment. This study was performed after the Ethics Committee for Animal Experimentation at Sinai University [SU.REC.2024 (33 A)] approval was granted (13 January 2025). The animals were arbitrarily allocated equally to 4 groups (n = 10); the control group received intraperitoneal (i.p.) saline injection once (15 mL/kg; i.p.). Diseased group: treated with a single i.p. injection of DOX at a dose of 15 mg/kg [47]. MB control group: received a daily dose of MB for 7 consecutive days (4 mg/kg; i.p.) [48]. Treatment group: rats received 4 mg/kg of MB daily for 7 consecutive days; the first dose of MB started after injection with DOX by one hour.

Doxorubicin (DOX) and methylene blue (MB) were administered intraperitoneally, a route commonly used in rodent studies to mimic systemic exposure comparable to intravenous administration in humans. This approach is widely accepted for evaluating cardiotoxicity and therapeutic modulation in preclinical models.

### 4.2. Sample Collection

Blood samples were drawn from the retro-orbital venous sinus of each animal into plain test tubes. After allowing 10 min for clotting, to separate the serum, the samples were centrifuged at 3000 rpm (4 °C) and then frozen at −80 °C for later examination of biochemical indicators. After the end of the treatment, rats received an intraperitoneal injection (1 mL) of anesthesia (0.3 mL of xylazine and 0.7 mL of ketamine) and were sacrificed using a sterile surgical blade. Prior to homogenization, one gram of hepatic tissue underwent triple washing with cold NaCl solution (0.9%) and was then homogenized in cold phosphate-buffered saline (PBS) (9 mL; pH 7.5) [49]. The hepatic homogenates were subjected to cold centrifugation for approximately 15 min at 3000 rpm, after which the supernatants were extracted to assess antioxidant, oxidative stress, and inflammatory levels. A small piece of liver was preserved in formalin for further histological and immunohistochemical examination.

### 4.3. Biochemical Analysis

#### 4.3.1. Serum Hepatic Markers

The serum aspartate aminotransferase (AST) and alanine aminotransferase (ALT) values were analyzed calorimetrically utilizing examining kits obtained from (Teco Diagnostics, Anaheim, CA, USA). Serum gamma glutamyl transferase (GGT) was evaluated utilizing a specific rat ELISA kit (My BioSource, San Diego, CA, USA, Cat #: MBS9343646). All techniques were performed according to the manufacturers’ protocols.

#### 4.3.2. Oxidative and Antioxidant Stress Markers

The homogenized liver samples were employed to elucidate the OS degree through measurement of the tissue concentrations of OH- radical using the specific ELISA kit (Enzo life science, Inc., 10 Executive Blvd, Farmingdale, NY, USA, Cat #: ADI-EKS-810A). The Rat Total Antioxidant Status (TAC) of the hepatic tissue samples was assessed using a quantification method with the specific ELISA kits (My BioSource, San Diego, CA, USA, Cat #: MBS1600693). The procedure was carried out according to the manufacturer’s protocols in the attached brochures.

TAC assay was performed to provide an integrated parameter reflecting the cumulative antioxidant defense status in cardiac tissue. This assay was selected because it evaluates the overall capacity of both enzymatic and non-enzymatic antioxidants, offering a broader picture of oxidative stress than measuring individual antioxidants alone. Given that oxidative damage plays a central role in doxorubicin-induced cardiotoxicity, TAC assessment directly supports this study’s aim of evaluating methylene blue’s potential cardioprotective and antioxidant effects.

#### 4.3.3. Estimation of Hepatic Tissue Concentrations of Apoptotic Biomarkers

The rat hepatic tissue levels of the apoptotic pathway markers PERK/GRP/CHOP were analyzed using specific rat ELISA kits purchased from My BioSource (San Diego, CA, USA, Cat #: MBS2511166) for PERK and from BT LAB (Shanghai, China) for GRP (Cat #:LS-F8683) and CHOP (Cat #: LS-F11285). The quantification method followed the manufacturers’ protocols.

### 4.4. Histopathological Examination

The hepatic tissues underwent preservation in 10% formalin solution before being subjected to conventional histological procedures. These procedures involved successive immersion in elevating ethanol concentration for dehydration, followed by paraffin wax embedding. Subsequently, the paraffin-embedded tissue blocks were sliced into 4 µm sections and stained using routine work stain, hematoxylin and eosin (H&E) [50].

### 4.5. Immunohistochemistry (IHC)

We used IHC to examine the presence and location of Caspase-3, NRF-2, P53, and NF-κB proteins in liver tissues. Sections were processed, and antigens were retrieved by boiling in citrate buffer (pH 6.0). Hydrogen peroxide (3%) was administered for 20 min to inhibit tissue endogenous peroxidase activity. Then, to avoid non-specific antibody interaction with tissue, 5% bovine serum albumin was employed as a blocking agent. Next, 4 µm liver sections were immunostained with anti-Caspase-3, NRF-2, P53, and NF-κB primary antibodies for 90 min [51]. Following that, the samples were incubated for 30 min with HRP-labeled 2^ry^ antibodies. The targeted proteins were stained with a diaminobenzidine (DAB) kit (ScyTek Laboratories, Inc., Logan, UT, USA), and hematoxylin was utilized as a counterstain. Tissue segment images were acquired with digital imaging equipment coupled to a light microscope (Leica Flexacam i5, Leica, Wetzlar, Germany). Signal intensity for Caspase-3, NRF-2, P53, and NF-κB immunoreactivity was determined in all groups at 400× magnification [52].

### 4.6. Assessment of IHC Staining

The quantitative IHC was performed by employing ImageJ Fiji software version 1.2. The intensity for positive Caspase-3, NRF-2, P53, and NF-κB immunoreactions was determined at magnification 400× for all groups [53].

### 4.7. Statistical Analysis

All data were gathered and analyzed with SPSS, version 22.0 (IBM Corporation, Armonk, NY, USA). Data were expressed as mean ± S.E.M. The distinction between groups was statistically examined using GraphPad Prism 5 (La Jolla, CA, USA) utilizing one-way ANOVA followed by the Tukey–Kramer Multiple Comparison Test. *p* values < 0.05 were considered significant.

## 5. Conclusions

MB exhibits remarkable hepatoprotective potency against DIHT. This protection is mediated via restoration of TAC; reduction of HO-1; suppression of ER stress signaling via downregulation of PERK, GRP78, and CHOP; and inhibition of apoptosis and inflammation through decreased p53, Caspase-3, and NF-κB expression. Additionally, partial restoration of Nrf2 activity reinforces antioxidant defenses, collectively preserving hepatocellular structure and function.

## Data Availability

The original contributions presented in this study are included in the article/Supplementary Material. Further inquiries can be directed to the corresponding author(s).

## Supplementary Materials

The following supporting information can be downloaded at: https://www.mdpi.com/article/10.3390/ph18111625/s1, Table S1. The animal body weight values throughout the experiment.
