# Supplementary material for: Hepatoprotection by Methylene Blue Against Doxorubicin Toxicity Through Coordinated Modulation of Oxidative Stress, ER Stress, and Apoptotic Pathways
Source: Pharmaceuticals (Basel). 2025 Oct 28;18(11):1625. doi: 10.3390/ph18111625 (PMC12655174; doi:10.3390/ph18111625)
Supplement: Supplementary file 1 [file pharmaceuticals-18-01625-s001.zip › pharmaceuticals-3853610-supplementary.pdf]

Table S1. The animal body weight vales throughout the experiment.

	Control		DOX		DOX+MB		MB	
	Initial	end	Initial	end	Initial	end	Initial	end
1	165	187	180	165	175	169	170	178
2	162	188	173	151	178	182	163	172
3	168	190	170	153	180	171	173	192
4	160	182	175	144	178	183	164	178
5	175	189	177	156	180	173	165	174
6	162	184	172	159	179	183	168	175
7	167	192	180	162	174	170	162	174
8	164	180	175	153	170	174	171	180
9	173	192	172	149	176	172	172	186
10	165	185	174	154	175	169	163	178
